# Advancing autonomy through lifelong learning: a survey of autonomous intelligent systems

**DOI:** 10.3389/fnbot.2024.1385778

**Published:** 2024-04-05

**Authors:** Dekang Zhu, Qianyi Bu, Zhongpan Zhu, Yujie Zhang, Zhipeng Wang

**Affiliations:** ^1^College of Electronic and Information Engineering, Tongji University, Shanghai, China; ^2^College of Science and Engineering, University of Glasgow, Glasgow, United Kingdom; ^3^College of Mechanical Engineering, University of Shanghai for Science and Technology, Shanghai, China

**Keywords:** artificial intelligence, lifelong learning, algorithm, autonomous intelligent systems, future perspectives

## Abstract

The combination of lifelong learning algorithms with autonomous intelligent systems (AIS) is gaining popularity due to its ability to enhance AIS performance, but the existing summaries in related fields are insufficient. Therefore, it is necessary to systematically analyze the research on lifelong learning algorithms with autonomous intelligent systems, aiming to gain a better understanding of the current progress in this field. This paper presents a thorough review and analysis of the relevant work on the integration of lifelong learning algorithms and autonomous intelligent systems. Specifically, we investigate the diverse applications of lifelong learning algorithms in AIS’s domains such as autonomous driving, anomaly detection, robots, and emergency management, while assessing their impact on enhancing AIS performance and reliability. The challenging problems encountered in lifelong learning for AIS are summarized based on a profound understanding in literature review. The advanced and innovative development of lifelong learning algorithms for autonomous intelligent systems are discussed for offering valuable insights and guidance to researchers in this rapidly evolving field.

## 1 Introduction

Autonomous intelligent systems (AIS), including intelligent robots, autonomous vehicles, and similar technologies, have emerged as a frontier direction in the field of artificial intelligence. These systems possess the ability to interact with humans and the environment, enabling them to execute tasks such as perception, planning, decision-making, and control. With the advancement of artificial intelligence, the algorithms employed by AIS for different tasks have transitioned from being model-driven to data-driven approaches. End-to-end AI algorithms based on deep learning, reinforcement learning, and other techniques have gained significant research attention.

However, as the data-driven algorithms rely on the type, scale, and quality of training data, the coherence, generality, and adaptability of the algorithms across different tasks and environments are great challenges. The challenge for AIS concerned in this paper is the ability to remember previous tasks when learning new ones, known as catastrophic forgetting (Shi et al., 2021). Catastrophic forgetting refers to the phenomenon where a neural network loses previously learned information after training on subsequent tasks, resulting in a drastic performance drop on previous tasks (Serra et al., 2018). Therefore, it is crucial to improve the capability of AIS for lifelong learning, which aims to enhance knowledge retention and transfer, thereby addressing the problem of catastrophic forgetting.

Lifelong learning algorithms have made significant progress in dealing with the core problems faced by AIS and mitigating the impact of catastrophic forgetting. Lifelong learning algorithms aim to sequentially acquire proficiency in multiple tasks while pursuing two primary objectives: ensuring that the acquisition of new tasks does not lead to catastrophic forgetting of previously learned knowledge (Zhou and Cao, 2021a), and leveraging prior task knowledge to facilitate the acquisition of novel tasks. Despite numerous achievements in lifelong learning in recent years, there are still evident shortcomings. Firstly, lifelong learning still heavily relies on labeling, which can be costly, troublesome, prone to errors, and impractical for providing persistent human labeling for all future tasks (He et al., 2021). Secondly, adapting to drift in adaptation spaces poses a challenge for lifelong learning. Drift in adaptation spaces arises from uncertainties that impact the quality properties of adaptation options, potentially leading to no adaptation option satisfying the initial set of adaptation goals, thereby damaging system quality (Gheibi and Weyns, 2023). Additionally, the big data problem presents another major challenge. AIS with lifelong learning algorithms must handle the continuous influx of changing data and adapt to learning problems effectively (Yang, 2013).

In this paper, we aim to provide a comprehensive overview of lifelong learning algorithms for autonomous intelligent systems, covering the recent development, related applications, and existing challenges that need to be addressed. Furthermore, we will discuss the future outlook of lifelong learning with autonomous intelligent systems. The main contributions of this paper are as follows:

1. The thoroughly review and analysis of AIS and lifelong learning, along with the rationale for combining these two fields, are introduced.
2. Relevant applications of lifelong learning algorithms with AIS are presented to showcase their significant role in different industry applications.
3. Remaining problems are analyzed, and academic insights into the future trends of AIS Lifelong learning are expounded.

The rest of the paper is organized as follows. Section II elucidates the background information on the emergence and historical milestones of AIS and lifelong learning. Section III presents various applications of lifelong learning algorithms with AIS, highlighting the research status and latest progress. In Section IV, A comprehensive review of issues and challenges in lifelong learning for AIS and the outlook and future trends are discussed. Finally, the main conclusions are given in Section V.

## 2 The developing lifelong learning and autonomous intelligent systems

### 2.1 Autonomous intelligent systems

In recent decades, remarkable progress has been made in the development of unmanned systems, ranging from robots to unmanned aerial vehicles (UAVs), unmanned ground vehicles (UGVs), and unmanned marine vehicles (UMVs). What once were programming-based systems have now transformed into automatic unmanned systems and are further advancing toward autonomous intelligent systems (AIS). AIS represents the forefront of artificial intelligence development, characterized by exceptional levels of autonomy and intelligence. By harnessing advanced technologies such as artificial intelligence (AI), big data, and robotics, AIS enables the execution of complex tasks and adaptive decision-making. This section explores the potential applications of AIS across various domains.

#### 2.1.1 Intelligent transportation and autonomous driving

The development of the automobile industry has driven an increased demand for safety and stability in modern transportation. As a result, autonomous driving technology has gained significant traction and is being widely deployed in the market (Xiao, 2022). This technology is revolutionizing intelligent transportation and smart city systems by enhancing the efficiency and safety of transportation networks. It’s worth noting that although autonomous driving has recently garnered more attention, the concept of autonomous vehicles dates back several decades, with various activities in this field taking place even further in the past (Khan, 2022).

The first autonomous car was introduced by Tsugawa at the Mechanical Engineering Laboratory in Tsukuba, Japan in the 1970s (OM Group of Companies, 2020). Subsequently, there have been numerous developments and initiatives worldwide. Notably, Ernst Dickmann’s vision guided Mercedes Benz in 1980 to achieve speeds of up to 39mph in a controlled environment (Delcker, 2020). With the integration of autonomous driving algorithms, vehicles possess self-navigating capabilities, real-time traffic monitoring, and adaptive route planning based on changing environmental conditions. Furthermore, autonomous driving vehicle enables the efficient management of traffic, congestion control, and the integration of advanced communication and information technologies, thereby facilitating intelligent infrastructure.

However, the utilization of autonomous driving faces significant challenges in complex traffic environments characterized by dynamic and variable scenarios. A key issue lies in perception algorithms encountering the long-tail problem, where rare or unforeseen events pose difficulties for standard algorithms to handle. This challenge becomes even more pronounced in mixed traffic scenarios involving both human-driven and autonomous vehicles. In such settings, algorithms must continually iterate and improve to adapt to the varying and unpredictable nature of the environment (Zhu et al., 2021; Zhou et al., 2022; Li et al., 2023). Therefore, lifelong learning is critical for the development of reliable and safe autonomous systems capable of operating effectively in real-world environments.

#### 2.1.2 Medical healthcare and service robotics

Service robots are typical AIS designed to assist humans, enhancing customer experiences across various industries such as hospitality, logistics, retail, and healthcare (Rajan and Cruz, 2022). With the advancements in AI and IoT technologies, service robots are continuously evolving and becoming more intelligent (Pan et al., 2010). The integration of healthcare and service robotics holds immense promise for improving patient care and enhancing efficiency. Intelligent service robots have the capability to assist in a range of tasks, including patient monitoring, medication dispensing, and patient support, thereby relieving healthcare professionals from repetitive and time-consuming responsibilities. Additionally, intelligent service robots can analyze medical data, provide personalized treatment recommendations, and contribute to remote healthcare services, leading to improved accessibility and quality of care. By leveraging the power of AIS, service robots in healthcare settings can not only streamline processes but also contribute to better patient outcomes. They serve as valuable tools in alleviating the burden on healthcare professionals, enabling them to focus on more complex and critical aspects of patient care. Moreover, AIS-driven analysis of medical data helps generate valuable insights that can inform decision-making and improve treatment strategies (Qu et al., 2021).

However, the integration of intelligent service robots in the field of healthcare also presents certain challenges. One significant challenge is ensuring the safety and reliability of these robots in critical medical environments. As they interact closely with patients, it is essential to address concerns regarding privacy, data security, and potential errors in their operations. Additionally, there is a need for standardized regulations and guidelines to govern the use of service robots in healthcare settings.

Moreover, the complexity and diversity of healthcare scenarios pose challenges for intelligent service robots. Medical environments can be unpredictable, requiring robots to adapt to various situations, handle unexpected events, and effectively communicate with both patients and healthcare professionals. Achieving seamless human-robot interaction and maintaining an appropriate balance between automation and human intervention is crucial in providing high-quality and patient-centric care.

#### 2.1.3 Urban security and UAV

UAV has garnered considerable attention in various military and civilian applications due to their improved stability and endurance (Mohsan et al., 2022). Over the past decade, UAVs have been employed in a wide range of fields, including target detection and tracking, public safety, traffic monitoring, military operations, hazardous area exploration, indoor and outdoor navigation, atmospheric sensing, post-disaster operations, health care, data-sharing, infrastructure management, emergency and crisis management, freight transport, wildfire monitoring and logistics (Hassija et al., 2019). For example, DARPA’s “Collaborative Operations in Denied Environment” (CODE) program seeks to enhance the mission capabilities of unmanned aerial vehicles (UAVs) by increasing autonomy and inter-platform collaboration. The United States military has integrated autonomous intelligent unmanned systems into combat through the Project Maven initiative, which employs artificial intelligence algorithms to identify relevant targets in Iraq and Syria. In the domain of urban security, UAV plays a critical role by leveraging AIS’s advanced surveillance and analytical capabilities. These intelligent drones enable efficient monitoring of public spaces, early detection of potential threats, and prompt response to emergencies. Moreover, AIS-driven drones enhance search and rescue operations, disaster management, and protection of critical infrastructure while minimizing human risk.

However, several crucial factors hinder the performance of UAVs in urban security. These factors include diverse scenes, stringent man–machine safety requirements, limited availability of training data, and small sample sizes (Carrio et al., 2017; Teixeira et al., 2023). Addressing these challenges is essential to ensure the optimal functioning of UAVs in urban security scenarios. Efforts should be made to develop robust and adaptable AI algorithms that can handle diverse environmental conditions encountered in urban settings. Additionally, ensuring the safety of UAV operations requires stringent regulations and standards for both hardware and software components. Acquiring more extensive and representative training datasets is also necessary to improve the accuracy and reliability of AI models used in UAV systems. Lastly, efforts should be made to address the limitations posed by small sample sizes by leveraging transfer learning techniques and collaborative data sharing initiatives.

#### 2.1.4 Ocean exploration and UMV

AIS contributes significantly to ocean exploration and research through the development of UMV equipped with advanced sensing and navigation capabilities. UMVs integrated with AI algorithms can be used for tasks such as scientific exploration, hydrological surveys, emergency search and rescue, and security patrols (Kingston et al., 2008; Wang et al., 2016). The Monterey Bay Aquarium Research Institute (MBARI) has significantly reduced the human resources required for data analysis by 81% and simultaneously increased the labeling rate tenfold through its Ocean Vision AI program, which trains a vast underwater image database. The autonomous underwater robot, CUREE, developed in collaboration with WHOI, can autonomously track and monitor marine animals, facilitating effective marine management. These wide-ranging applications have contributed to the development of motion control techniques and have produced many interesting results in the literature, such as heading control (Kahveci and Ioannou, 2013), trajectory tracking control (Katayama and Aoki, 2014; Ding et al., 2017), formation control (Li et al., 2018; Liao et al., 2024), and path-following problems (Shen et al., 2019).

The ocean environment presents complex and variable challenges that demand adaptive capabilities from UMV. In the deep-sea environment, UMV encounter various challenges, including changes in underwater terrain, marine biodiversity, and ocean currents. These changes can result in variations in sensor data and diverse appearances of targets. By employing lifelong learning algorithms, unmanned systems can adapt and learn in real-time, enhancing their performance and robustness (Wibisono et al., 2023). Furthermore, deep-sea environments pose limitations in communication bandwidth, latency, and mission execution times. Traditional machine learning algorithms often struggle to adapt to new environments and tasks, as they are typically trained for specific purposes. Lifelong learning algorithms offer a solution by reducing reliance on external resources and human intervention. UMV equipped with these algorithms can autonomously learn and make decisions, increasing their independence and reliability (Wang et al., 2019).

#### 2.1.5 Deep space exploration and spacecraft

Intelligent or autonomous control of an unmanned spacecraft is a promising technology (Soeder et al., 2014). And the ground-based mission control center will no longer be able to help the astronauts diagnose and fix spacecraft issues in real-time due to the longer connection durations associated with deep space exploration, using lifelong learning algorithms, unmanned systems can accumulate experience and knowledge during task execution and reduce reliance on frequent interactions and updates, enhancing their autonomy and adaptability (Jeremy and et.al, 2013). Also, the deep space environment is extremely complex and full of unknown and uncertain factors, such as the landform of the planet’s surface, the relationship between celestial bodies, and the atmosphere of the planet. Traditional machine learning algorithms are difficult to pre-train to adapt to all possible situations. Lifelong learning algorithms enable unmanned systems to constantly learn and adapt to new environments and tasks as they explore (Bird et al., 2020). What is more, in deep space exploration missions, unmanned systems typically need to process huge data streams from various sensors and extract useful information from them. Lifelong learning algorithms can help systems automatically discover and learn new features and patterns, thereby improving their perception and understanding (Choudhary et al., 2022). As a result, each vehicle core subsystem will contain inbuilt intelligence to allow autonomous operation for both normal and emergency operations including defect identification and remediation. This extends previous work on creating an autonomous power control (Soeder et al., 2014) which involves the development of control architectures for deep space vehicles (Dever et al., 2014; May et al., 2014) and using software agents (May and Loparo, 2014). As a result, the application of AIS in deep space exploration and spacecraft missions opens up new frontiers for scientific discovery. Intelligent spacecraft equipped with AIS can autonomously navigate, perform complex maneuvers, and adapt to dynamic space environments. Advanced AI-based algorithms enable real-time analysis of vast amounts of space data, autonomous targeting, and intelligent resource allocation, facilitating enhanced mission efficiency and enabling breakthrough discoveries.

In conclusion, the development of unmanned systems has evolved from programming-based to AIS. AIS leverages advanced technologies such as AI, big data, and robotics to enable complex tasks and adaptive decision-making. Across domains including intelligent transportation, healthcare, urban security, ocean exploration, and space missions, AIS demonstrates immense potential for revolutionizing various industries and pushing the boundaries of technological advancements. However, Autonomous intelligent systems require continuous learning to enable their applications in various domains. With the advancements in technologies such as deep learning, reinforcement learning, and large-scale AI models like AIGC (Artificial Intelligence General Cognitive), AISs are moving toward achieving general task learning and lifelong evolution. Establishing a lifelong learning paradigm is crucial for the future development of these autonomous systems. Embracing this paradigm will pave the way for remarkable advancements in the field of autonomous intelligent systems.

Besides the technical perspective, there are actually other angles people should take into consideration to enrich and improve the connotation of autonomous intelligent systems. For one thing, the ethical and social perspective cannot be ignored. Ethically and socially, the deployment of autonomous intelligence systems raises significant questions around accountability, privacy, job displacement, and fairness. The decision-making processes of AIS need to be transparent, explainable, and align with societal values to ensure trust and acceptance. Addressing these concerns involves interdisciplinary research, incorporating insights from ethics, law, and social sciences into the development and governance of AIS. For another thing, autonomous intelligent systems are also closely linked to the Sustainable Development Goals. They have the potential to help address global challenges in environmental protection, health, education and more, such as protecting the environment through intelligent monitoring and management of resources, or improving the quality and accessibility of education through personalized education systems. However, this also requires environmental impact, resource consumption and long-term sustainability to be taken into account when designing and applying autonomous intelligent systems.

### 2.2 Lifelong learning

Lifelong learning, alternatively known as continuous learning or incremental learning, traces its roots back to the mid-20th century. Early computer scientists and artificial intelligence researchers contemplated ways to enable computer systems to continuously learn and adapt to new knowledge. The adage “one is never too old to learn” holds true and applies equally to AIS.

**Figure 1:**
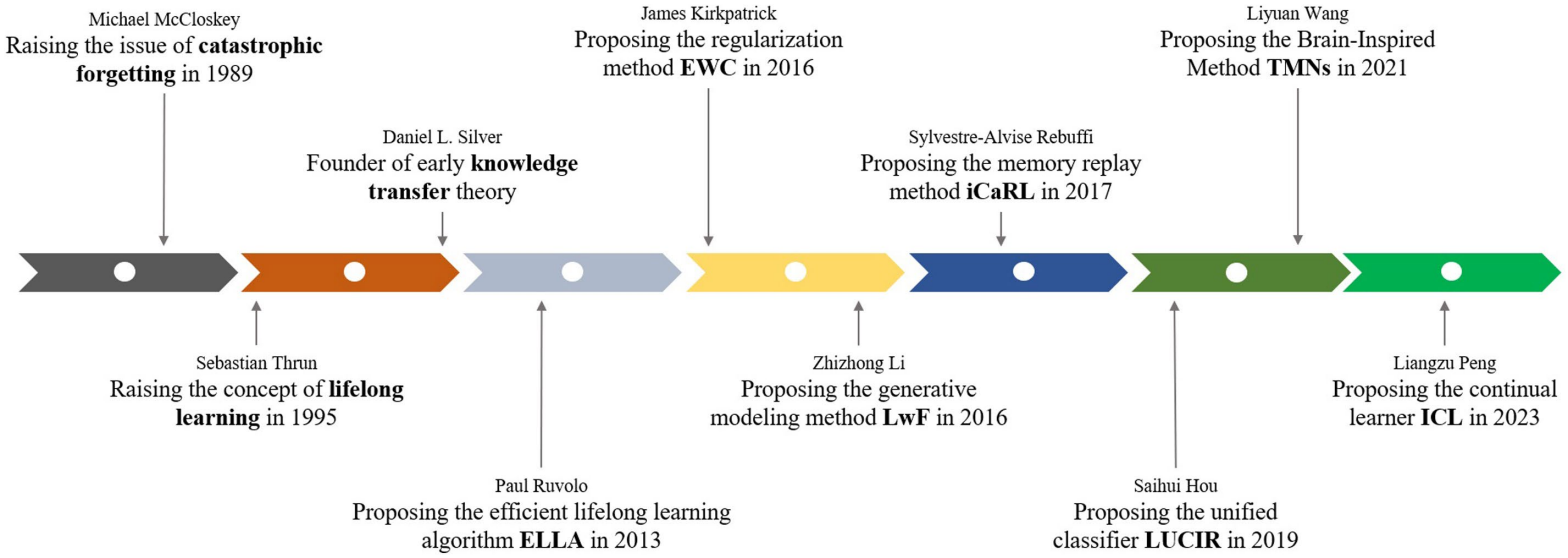
The history of the development of lifelong learning.

In 1957, Frank Rosenblatt’s perceptron emerged as an early neural network model that introduced the idea of machines improving their ideas and performance gradually through repeated training (Block et al., 1962). The era of artificial intelligence algorithms based on neural networks was begun. But for a long time, neural networks could not handle multiple tasks, nor could they handle dynamic tasks of time series. During the 1990s, the concept and research of transfer learning started to develop, positively influencing the notion of lifelong learning. Transfer learning focused on leveraging previously acquired knowledge for new tasks (Pan et al., 2010). In the 2000s, incremental learning began to emerge in lifelong learning research, enabling AI systems to learn new tasks without sacrificing previously acquired knowledge (Zhou et al., 2022). This approach helps in continuously improving the AI system’s performance, adapting to changes in the data distribution, and avoiding catastrophic forgetting. Incremental learning is particularly useful in dynamic environments where new data arrives regularly and the model needs to be continuously updated to maintain its accuracy and relevance. In our dynamically changing world, where new classes appear frequently, fresh users in the authentication system and a machine learning model ought to identify new classes while not forgetting the memory of previous ones (Zhou et al., 2022). If the dataset of old classes is no longer available, directly fine-tuning a deployed model with new classes might bring about the so-called catastrophic forgetting problem in which information about past classes is quickly forgotten (Hinton et al., 2015; Kirkpatrick et al., 2017; Shin et al., 2017). Hence, incremental learning, a framework that enables online learning without forgetting, has been actively investigated (Kang et al., 2022). From the 2000s to 2020s, Researchers have proposed various incremental learning algorithms and techniques to address the challenges associated with learning from evolving data. These algorithms focus on updating the model efficiently (Lv et al., 2019; Tian et al., 2019; Zhao et al., 2021; Ding et al., 2024), handling concept drift (Schwarzerova and Bajger, 2021), managing memory constraints (Smith et al., 2021), and balancing stability and plasticity in the learned knowledge (Wu et al., 2021; Lin et al., 2022; Kim and Han, 2023). Additionally, incremental learning has been explored in different domains, including image classification (Meng et al., 2022; Nguyen et al., 2022; Zhao et al., 2022), natural language processing (Jan Moolman Buys University College University of Oxford, 2017; Kahardipraja et al., 2023), recommender systems (Ouyang et al., 2021; Wang et al., 2021; Ahrabian et al., 2021a), and data stream mining (Eisa et al., 2022). Researchers have investigated different strategies such as incremental decision trees (Barddal and Fabr’ıcio Enembreck., 2020; Choyon et al., 2020; Han et al., 2023), online clustering (Bansiwala et al., 2021), ensemble methods (Lovinger and Valova, 2020; Zhang J. et al., 2023), and deep learning approaches to tackle incremental learning problems (Ali et al., 2022). Incremental learning enables lifelong learning to constantly learn new data new data while leveraging prior knowledge that continues to be an active research topic (Figure 1).

Lifelong learning plays a crucial role in enhancing the performance of Artificial Intelligence Systems (AIS) due to its powerful capabilities. It enables AIS to continuously update their knowledge and skills, allowing them to effectively handle consecutive tasks in dynamic and evolving environments.

There are three main research methods used in lifelong learning:

- Regularization-based Approach: This method consolidates past knowledge by incorporating additional loss terms that reduce the rate of learning for important weights used in previously learned tasks. By doing so, it minimizes the risk of new task information significantly altering the previously acquired weights (Shaheen et al., 2022). An example of this approach is Elastic Weight Consolidation (EWC), which penalizes weight changes based on task importance, regularizing model parameters and preventing catastrophic forgetting of previous experiences (Febrinanto et al., 2022).
- Rehearsal-based Approach: This method focuses on preserving knowledge by leveraging generative models to replay tasks whenever the model is modified or by storing samples from previously learned tasks in a memory buffer (Faber et al., 2023). One notable approach is Prototype Augmentation and Self-Supervision for Incremental Learning (PASS) (Zhu et al., 2021).
- Model-based Approach: To prevent forgetting, models can be expanded to improve performance, or different models can be assigned to each task. Examples of this approach include Packnet (Mallya and Lazebnik, 2018a) and Dynamically Expandable Representation for Class Incremental Learning (DER) (Yan et al., 2021).

These research methods offer distinct strategies for addressing the challenges associated with lifelong learning in the context of handling consecutive tasks in dynamic and evolving environments. The choice of the most suitable approach depends on specific requirements and circumstances. Ongoing research in the field of lifelong learning continues to explore innovative techniques and approaches to further enhance the performance and adaptability of AIS.

However, the combination of lifelong learning and autonomous intelligent systems poses several challenges due to perceptual cognitive algorithms (Nicolas, 2018; Hadsell et al., 2020), varying tasks (Kirkpatrick et al., 2017; Aljundi et al., 2021), changing environments (Zenke et al., 2017a), and limitations in computing chips (Mallya and Lazebnik, 2018b), control systems (Kober et al., 2013; Andrei et al., 2017), and the diverse range of system types (Kemker and Kanan, 2017; Parisi et al., 2017). Currently, research on this integration is insufficient, and numerous difficulties remain to be addressed. Among these challenges, catastrophic forgetting is a prominent problem wherein previously learned tasks may be forgotten when AIS learns new ones. Consequently, solving this problem holds immense significance and remains a core objective of lifelong learning.

There are three main dimensions to handle catastrophic forgetting:

#### 2.2.1 Knowledge retention

If there is only one model continuously learning different tasks, we naturally expect it not to forget knowledge previously learned when it learns new tasks. In addition, the model is supposed to prevent stopping learning just in order to retain what has been learned at the same time. There are several methods such as Elastic Weight Consolidation (EWC) (Aich, 2021), Synaptic Intelligence (SI) (Zenke et al., 2017b), Memory Aware Synapses (MAS) (Aljundi et al., 2018).

#### 2.2.2 Knowledge transfer

It is expected that models are able to utilize what they have learned to help handle new problems. Related method is Gradient Episodic Memory (GEM) (Lopez-Paz and Ranzato, 2022).

#### 2.2.3 Model expansion

Sometimes, models may be too simple to handle complicated tasks, so it is expected that these models could expand themselves to more complicated ones according to the complexity of problems. Some related methods are Progressive Neural Networks (Rusu et al., 2022), Expert Gate (Aljundi et al., 2017), Net2Net (Chen et al., 2016; Sodhani et al., 2019).

## 3 Representative applications of lifelong learning for AIS

**Figure 2:**
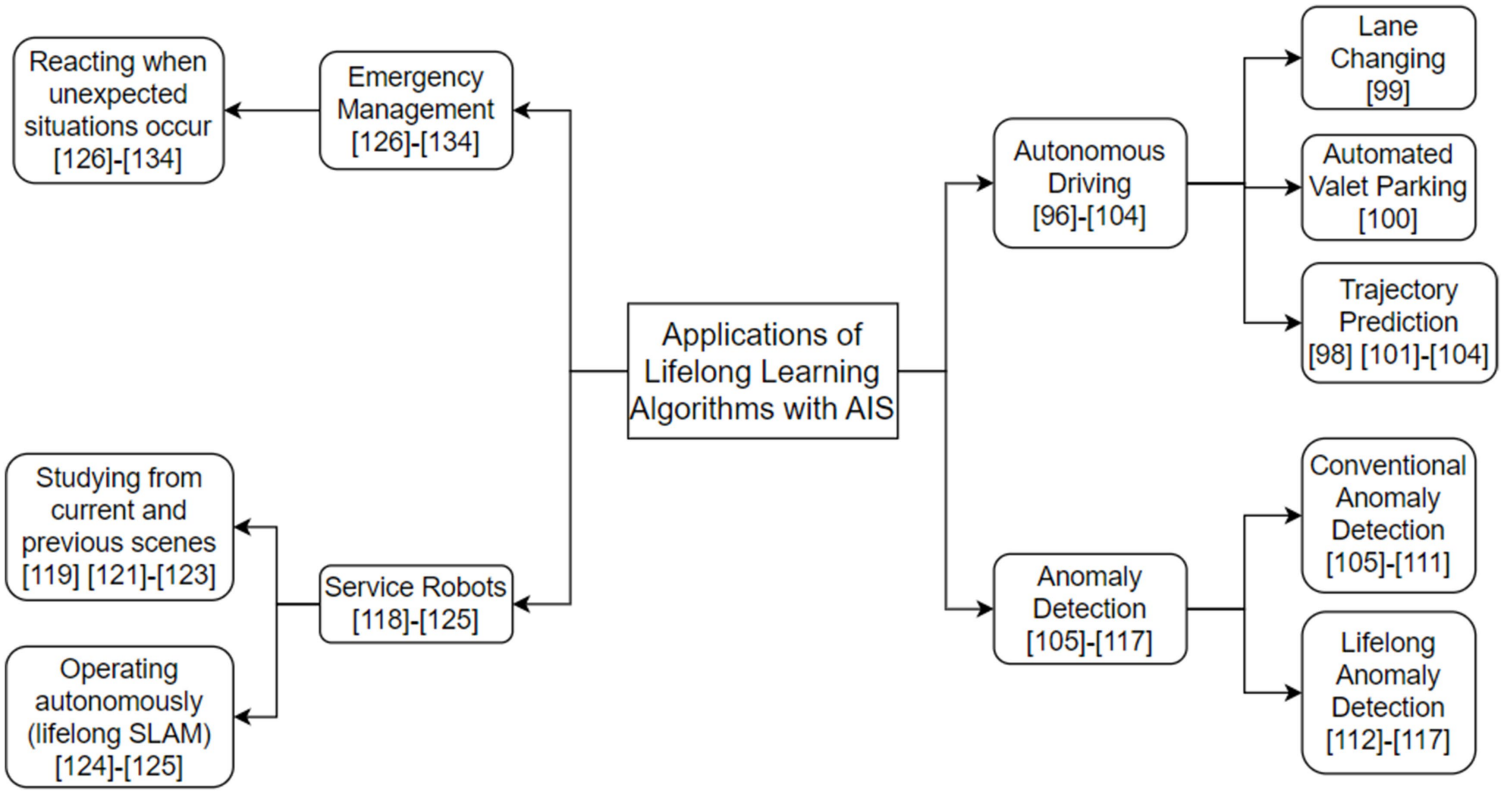
The taxonomy of technologies related to applications of lifelong learning algorithms for AIS.

Nowadays, it is an increasingly popular trend to use lifelong learning algorithms for AIS, which could better improve the performance of these systems. There have been plenty of domains making use of lifelong learning algorithms, here we highlight some representative and contemporary examples below (Figure 2).

### 3.1 Autonomous driving

The development of autonomous vehicles has advanced quickly in recent years (Han et al., 2023). Modern vehicles are becoming more and more automated and intelligent due to advancements in lifelong learning algorithms, mechanical, and computing technologies (Su et al., 2012). The Institute of Electrical and Electronics Engineers (IEEE) alone produced around 43,000 conference papers and 8,000 journal (including magazine) articles on the subject of autonomous driving in the 5 years between 2016 and 2021 (Chen et al., 2022). Many IT and automotive companies have been attracted to this promising field, such as Baidu Apollo, Google Waymo. And by 2021, Waymo’s autonomous vehicles have driven more than 20 million miles on the road, demonstrating the reliability and safety of the technology of autonomous driving. As a result, in the near future, different types of AVs are expected to be fully commercialized, with a significant impact on all aspects of our lives (Su et al., 2012).

The most challenging problem autonomous driving currently faces is to adapt to novel driving scenarios, especially in complex and mixed traffic environments, and react properly and rapidly in time. As a result, autonomous driving is particularly in need of the combination of lifelong learning algorithms. So in the section below, different frames of lifelong learning in some crucial fields of autonomous driving are explained.

#### 3.1.1 Lane changing

Lane changing is one of the largest challenges in the high-level decision-making of autonomous vehicles (AVs), especially in mixed and dynamic traffic scenarios, where lane changing has a significant impact on traffic safety and efficiency. In recent years, the application of lifelong learning to lane-changing decision-making in AVs has been widely explored with encouraging results. However, most of these studies have focused on single-vehicle environments, and lane-changing in situations where multiple AVs coexist with human-driven vehicles has received little attention (Zhou et al., 2022), which should be paid more attention. In this regard, Ref. (Zhou et al., 2022) proposes a multi-agent advantage actor-critic method which uses a novel local reward design and parameter sharing scheme to formulate the lane changing decision of multiple AVs in a mixed traffic highway environment as a multi-agent lifelong learning problem using a lifetime learning algorithm.

#### 3.1.2 Automated valet parking

Automated valet parking (AVP) allows human drivers to park their cars in a drop-off zone (e.g., a parking garage entrance). These cars can independently perform autonomous driving tasks from the parking area to a designated parking space. AVP can greatly improve driver convenience, and is seen as an entry point for the promotion of AVs. And high-precision indoor positioning service is unavoidable in AVP. However, existing wireless indoor positioning technologies, including Wi-Fi, Bluetooth, and ultra-wideband (UWB), have a tendency to degrade significantly with the increase of working time and the change of building environments (Zhao et al., 2023). To handle this problem, a data-driven and map-assisted indoor positioning correction model has been proposed to improve the positioning accuracy for the infrastructure-enabled AVP system recently by a research team from Tongji University, Shanghai, China (for details refer to Ref. (Zhao et al., 2023)). In order to sustain the lifelong performance, the model is updated in an adversarial manner using crowdsourced data from the on-board sensors of fully instrumented autonomous vehicles (Zhao et al., 2023).

#### 3.1.3 Trajectory prediction

Accurate trajectory prediction of vehicles is the key to reliable autonomous driving. Adapting to changing traffic environments and implementing lifelong trajectory prediction models are crucial in order to maintain consistent vehicle performance across different cities. In real applications, intelligent vehicles equipped with autonomous driving systems should travel on different roadways, cities and even countries. The system needs to properly forecast the future trajectories of the surrounding vehicles and adapt to the diverse distribution of their motion and interaction pattern in order to safely guide the vehicle. In order to achieve this, the system must constantly acquire new information about developing traffic conditions while retaining its previous understanding. Furthermore, the system cannot afford to store a significant amount of trajectory data due to its restricted storage resources (Bao et al., 2021). So, in order to perform well on all processed tasks, it is necessary to keep lifelong learning with restricted storage resource. As a consequence, in a bid to achieve lifelong trajectory prediction, a new framework based on conditional generative replay is proposed by the research team from the University of Science and Technology of China (USTC), which handles the problem of catastrophic forgetting due to different types of traffic environments and improve the precision and efficiency of vehicle trajectory prediction (Bao et al., 2021).

At the moment, autonomous vehicles are not perfect in their operation (Chen et al., 2022), as evidenced by some accidents caused by autonomous driving vehicles in recent years, in which safety drivers were unable to prevent the accidents from occurring, resulting in the loss of multiple lives, thus bringing about these mournful aftermaths which could have been prevented. Obviously, in terms of performance, autonomous vehicle systems are still far from the visual systems of humans or animals (Chen et al., 2020). It is necessary to find novel solutions, such as bio-inspired visual sensing, multi-agent collaborative perception, and control capabilities that emulate biological systems’ operational principles (Tang et al., 2021). It is predicted that after reaching increasing degrees of robotic autonomy and vehicle intelligence, autonomous driving will become sufficiently safe and dependable by 2030 to replace the majority of human driving (Litman, 2021).

### 3.2 Anomaly detection

Anomaly detection is the task of finding anomalous data instances, which therefore represents deviations from the normal conditions of a process (Aggarwal, 2017). In many fields and real-world applications, such as network traffic invasions (Faber et al., 2021), aberrant behavior in cyber-physical systems like smart grids (Corizzo et al., 2021), or flaws in manufacturing processes (Alfeo et al., 2020), the ability to identify abnormal behavior is crucial.

Examples of relevant techniques for detecting anomalies in one-class learning are: (i) Autoencoder, a model based on neural network reconstruction; (ii) One-Class Support Vector Machine, which provides anomaly scores by contrasting new data with the decision boundary based on hyperplanes.; (iii) Local Outlier Factor, which provides an anomaly score that is derived from the ratio of the new data samples’ local density to the average local density of its closest neighbors; (iv) Isolation Forest, which offers tree ensembles and calculates the new samples’ anomaly score by measuring the distance from the root to the leaf; (v) Copula-based anomaly detection, which draws conclusions about the level of “extremeness” of data samples by using tail probabilities (Goldstein and Uchida, 2016; Li et al., 2020; Lesouple et al., 2021).

However, although these methods have been established and perform well in many scenarios, due to the catastrophic forgetting, the performance of the anomaly detection system is affected negatively when previous circumstances reoccur. For this reason, lifelong anomaly detection is supposed to be applied to balance between knowledge transferring and knowledge retention. Since many real-world domains are characterized by both recurrent conditions and dynamic, rapidly evolving situations, lifelong anomaly detection may out to be quite advantageous in these kinds of environments. This feature necessitates model characteristics that promote concurrent learning and adaptability (Faber et al., 2023). And several recent research efforts have begun to address the problem of lifelong anomaly detection. Examples include using meta-learning to estimate parameters for numerous tasks in one-class image classification (Frikha et al., 2021), transfer learning for anomaly detection in videos (Doshi and Yilmaz, 2020), and change-point detection in conjunction with memory arrangement (Corizzo et al., 2022). Particularly, in the field of autonomous driving, an effective collaborative anomaly detection methodology known as ADS-Lead was proposed to safeguard the lane-following mechanism of ADSs. It has a unique transformer-based one-class classification algorithm to detect adversarial image examples (traffic sign and lane identification threats) as well as time series anomalies (GPS spoofing threat) (Han et al., 2023). In addition, to enhance the anomaly detection performance of models, an active lifetime anomaly detection framework was provided for class-incremental scenarios that supports any memory-based experience replay mechanism, any query strategy, and any anomaly detection model (Faber et al., 2022).

**Figure 3:**
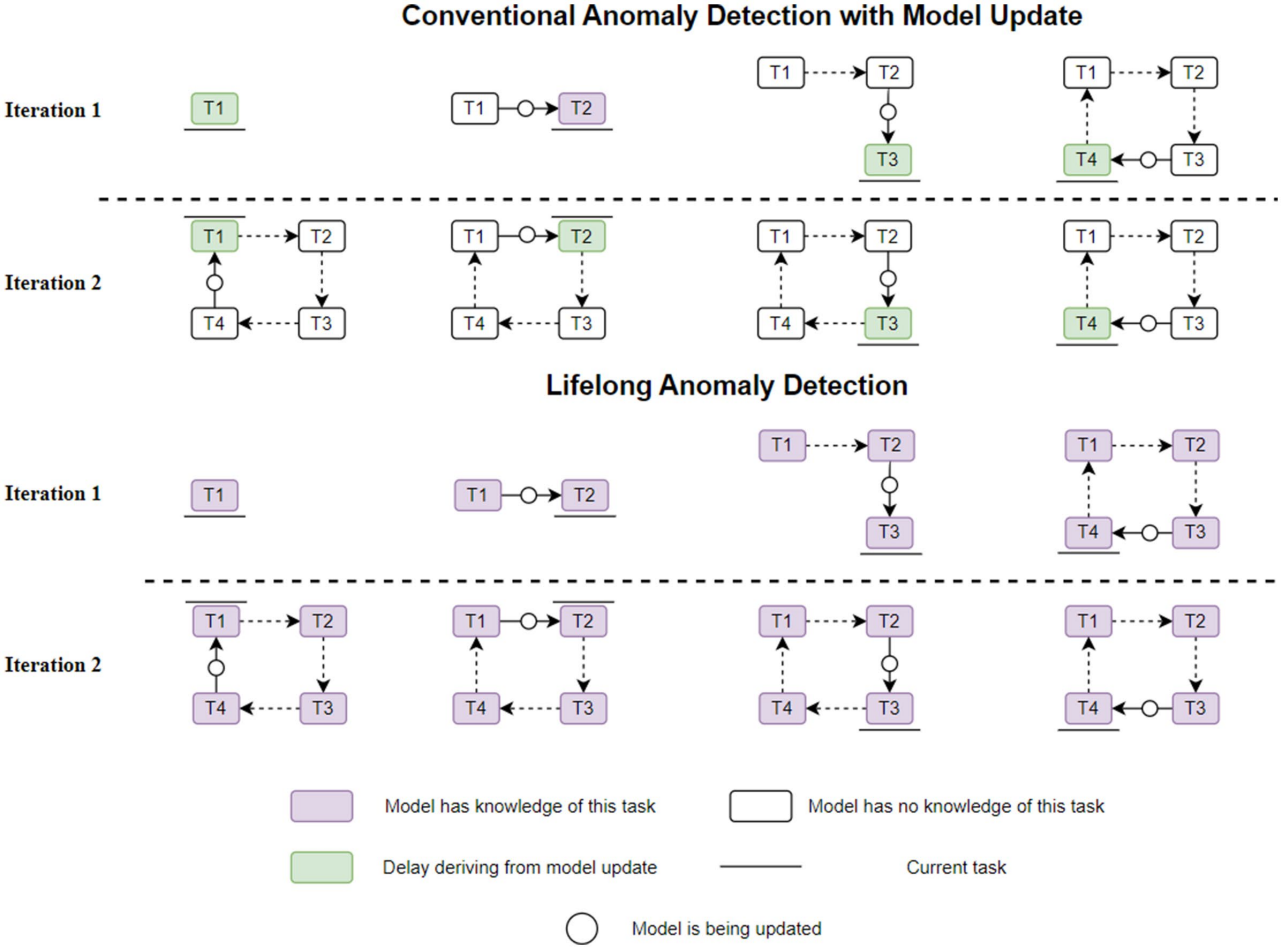
A scenario with four recurring tasks (Comparisons between conventional and lifelong anomaly detection).

Figure 3 illustrates a typical scenario comparing conventional anomaly detection with model updating with lifelong anomaly detection. In contrast to conventional anomaly detection, which continuously updates the model and causes detection delays, or false predictions, until the new task is incorporated into the model, lifetime anomaly detection in the second iteration does not require model updates following a recurrence of each work. Furthermore, in a 100-iteration scenario, only 4 model updates would be needed for lifetime anomaly detection, as opposed to 400 model updates for traditional anomaly detection, which results in detection delays. It could be used to map a wide range of recurring real-world scenarios, such as human activity sequences, geophysical phenomena like weather patterns, and cyber-physical system operating conditions (Faber et al., 2023; Figure 3).

**Figure 4:**
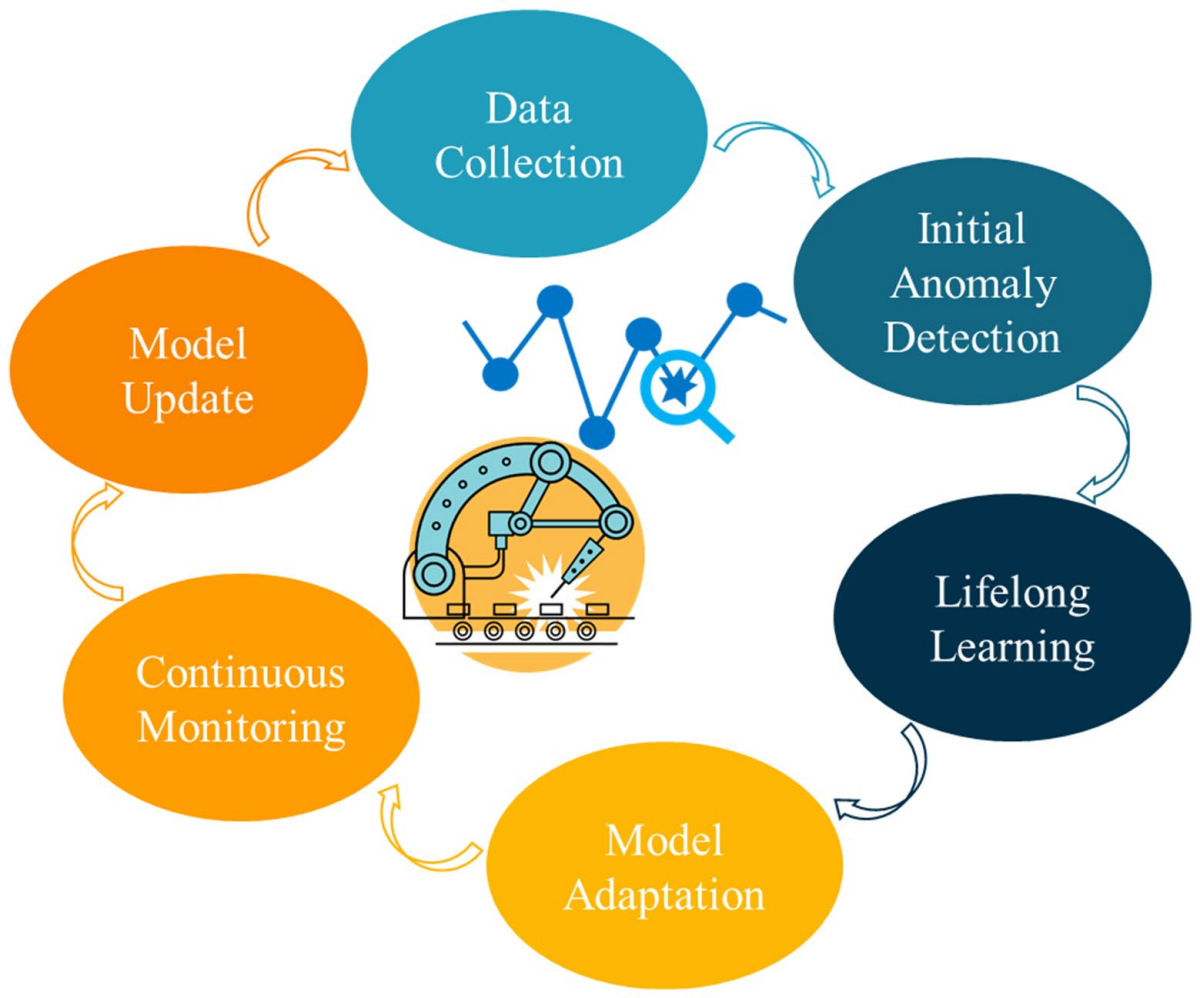
The core process of lifelong anomaly detection.

The core process of lifelong anomaly detection involves several key steps, as depicted in Figure 4. These steps include data collection, initial anomaly detection, lifelong learning, model adaptation, continuous monitoring, model update, and the repetition of the process. The first step is data collection, wherein data is gathered from multiple sources, such as network traffic, smart grids, and manufacturing processes. Following data collection, initial anomaly detection techniques, such as Autoencoders, Support Vector Machines, Local Outlier Factor, and Isolation Forests, are employed to conduct preliminary anomaly detection. Subsequently, lifelong learning takes place, whereby new data is integrated into the model while existing knowledge is updated and retained. Model adaptation is then performed based on the new data, which may involve applying techniques like meta-learning, transfer learning, or change point detection with memory organization. Continuous monitoring of the data for anomalies is carried out to ensure timely detection. To maintain the model’s effectiveness, periodic model updates are performed by refreshing it with new data and employing advanced techniques. This entire process is repeated cyclically, encompassing both data collection and model updating stages.

### 3.3 Service robots

Depending on the continuous learning mechanism for a variety of various robotic tasks, lifelong machine learning has drawn intriguing academic interests in the field of robotics (Dong et al., 2022). And past research has identified lifelong learning as a critical capability for service robots. Creating an artificial “lifelong learning” agent that can construct a cultivated understanding of the world from the current scene and their prior knowledge through an autonomous lifelong system is one of the big ambitions of robotics (She et al., 2020). According to a report by Allied Market Research, the global service robot market is valued at $21.084 billion in 2020 and is expected to reach $293.087 billion by 2032, with a CAGR of 24.3% from 2023 to 2032. Moreover, the number of new startups named after service robots accounts for 29% of all U.S. robotics companies. Those data, among other similar figures, remark the development in the service robots area (Gonzalez-Aguirre et al., 2021). Service robots are mostly tasked with helping humans in the home environment, and they must handle a wide variety of objects. These objects are dependent on the particular environment (e.g., bedroom, toilet, balcony), the human being supported (e.g., kids, elderly people, disabled people). It is practically impossible to prepare all possible objects at the time of or prior to the deployment of the robot. Therefore, the robots will need to adjust to new objects and different ways of perceiving things throughout their lives (Niemueller, 2013). Despite these challenges, we want these robots to notice us and show adaptive behavior when they are on a mission. When a robot is given negative feedback when vacuuming while someone is watching TV, it should be able to recognize this as a new context and adjust its behavior accordingly in similar spatial or social contexts. For example, when people are reading books, the robot should be able to connect this scenario to the one it has previously encountered and cease vacuuming (Irfan et al., 2021). Another example is when service robots engage in language teaching, they may encounter variations in language environments and user learning needs. In such cases, it is imperative for service robots to achieve self-learning and improvement by monitoring user feedback, autonomously exploring language environments, and utilizing natural language processing techniques. Only through these means can they better provide personalized language learning support and practical opportunities for users, thus enhancing teaching proficiency and efficiency (Kanero et al., 2022).

Another aspect of lifelong learning applied to robots is the ability to function independently for lengthy periods of time in dynamic, constantly-changing surroundings. For example, in a domestic scene, where most objects are likely to be movable and interchangeable, the visual character of the same place may differ markedly over successive days. To deal with this situation, a term lifelong SLAM has been in use to address SLAM problems in environments that have been changing over time, improving the robustness and accuracy of pose estimation of robots (Shi et al., 2020). Lifelong SLAM takes into account a robot’s long-term operations, which involve repeatedly visiting previously mapped places in dynamic surroundings. In lifetime SLAM, we make the assumption that a region is constantly mapped over an extended period of time, rather than only once (Kurz et al., 2021). Compared to classical SLAM methods, however, there exist a lot of challenges (Shi et al., 2020):

- Changed viewpoints - the robot may look at the same scene or items from several angles.
- Changed things - the objects may have been changed when reentering a place that was previously observed by the robot.
- Changed illumination - there could be a significant change in illumination.
- Dynamic objects - There could be objects in the scene that are moving or changing.
- Degraded sensors - unpredictable sensor noises and calibration errors could result from a variety of factors, including mechanical strain, temperature changes, dirty or damp lenses, etc.

**Figure 5:**
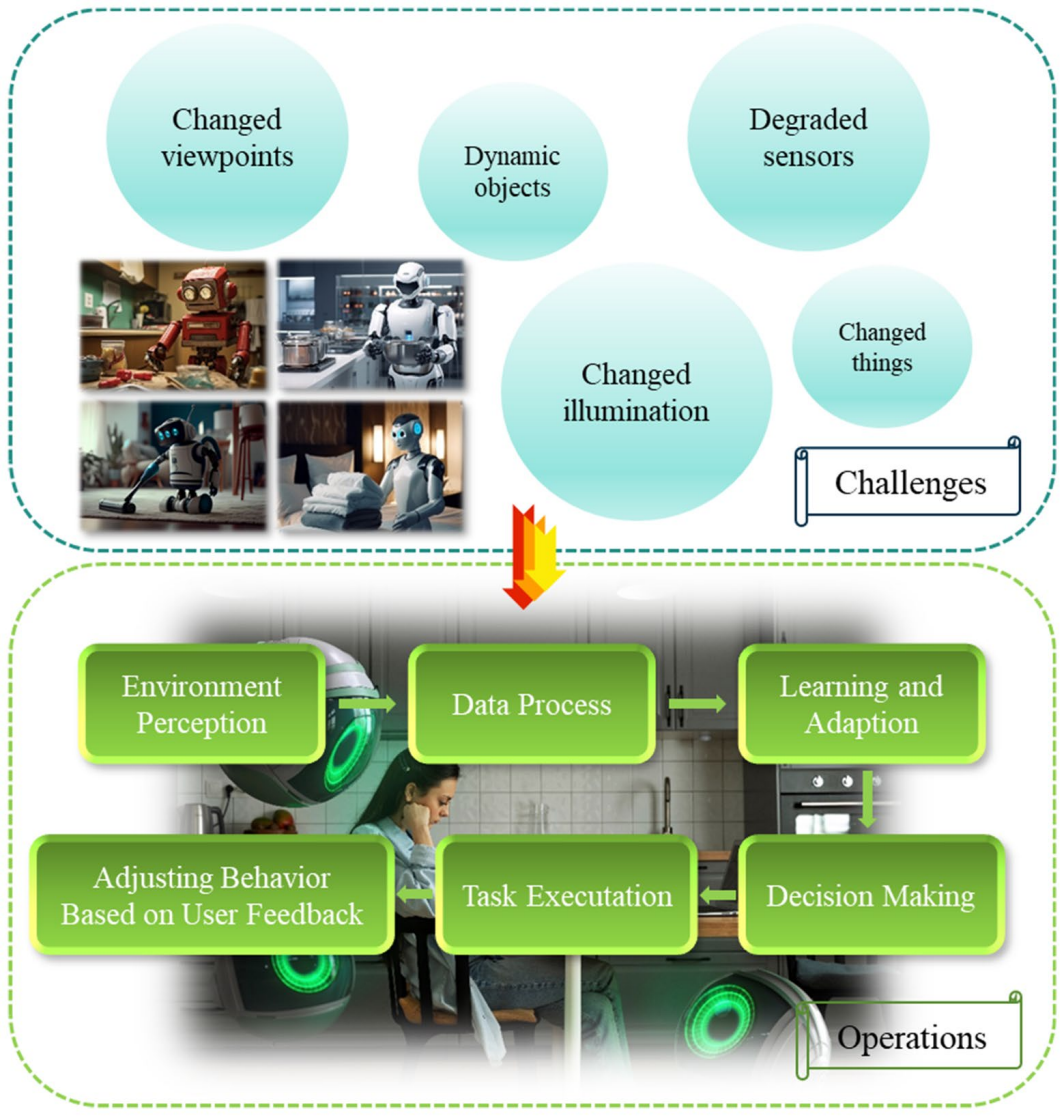
The operational flow of the lifetime service robot.

To address these challenges, the operational flow of the lifetime service robot is shown in Figure 5.

### 3.4 Emergency management

In recent years, machine learning algorithms have made great strides in enabling autonomous agents to learn through observation and sensor feedback how to carry out tasks in complex online environments. In particular, recent developments in deep neural network-based lifelong learning have demonstrated encouraging outcomes in the creation of autonomous agents that can interact with their surroundings in a variety of application domains (Arulkumaran et al., 2017), including learning to play games (Brown and Sandholm, 2017; Xiang et al., 2021), generating optimal control policies for robots (Jin et al., 2017; Pan et al., 2017), natural language processing and speech recognition (Bengio et al., 2015), body emotion understanding (Sun and Wu, 2023), as well as choosing the best trades in light of the shifting market conditions (Deng et al., 2017). The agent gradually learns the best course of action for the assigned task by seeing how its actions result in rewards from these encounters.

These methods are effective when it can be presumed that every event that occurs during deployment is a result of the same distribution that the agent was trained on. However, agents that must operate for extended periods of time in complex, real-world environments may be subject to unforeseen circumstances beyond the distribution for which they were designed or trained, due to changes in the environment. For instance, a construction site worker may unintentionally place a foreign object—like their hand–inside the workspace of a vision-guided robot arm, which must then react to prevent harm or damage. Similarly, an autonomous driving car may come across significantly distorted lane markings that it has never encountered before and must decide how to continue driving safely. In such unexpected and novel situations, the agent’s strategy will not apply, leading to the possibility of the agent taking unsafe actions. And that is what makes emergency management crucial.

**Figure 6:**
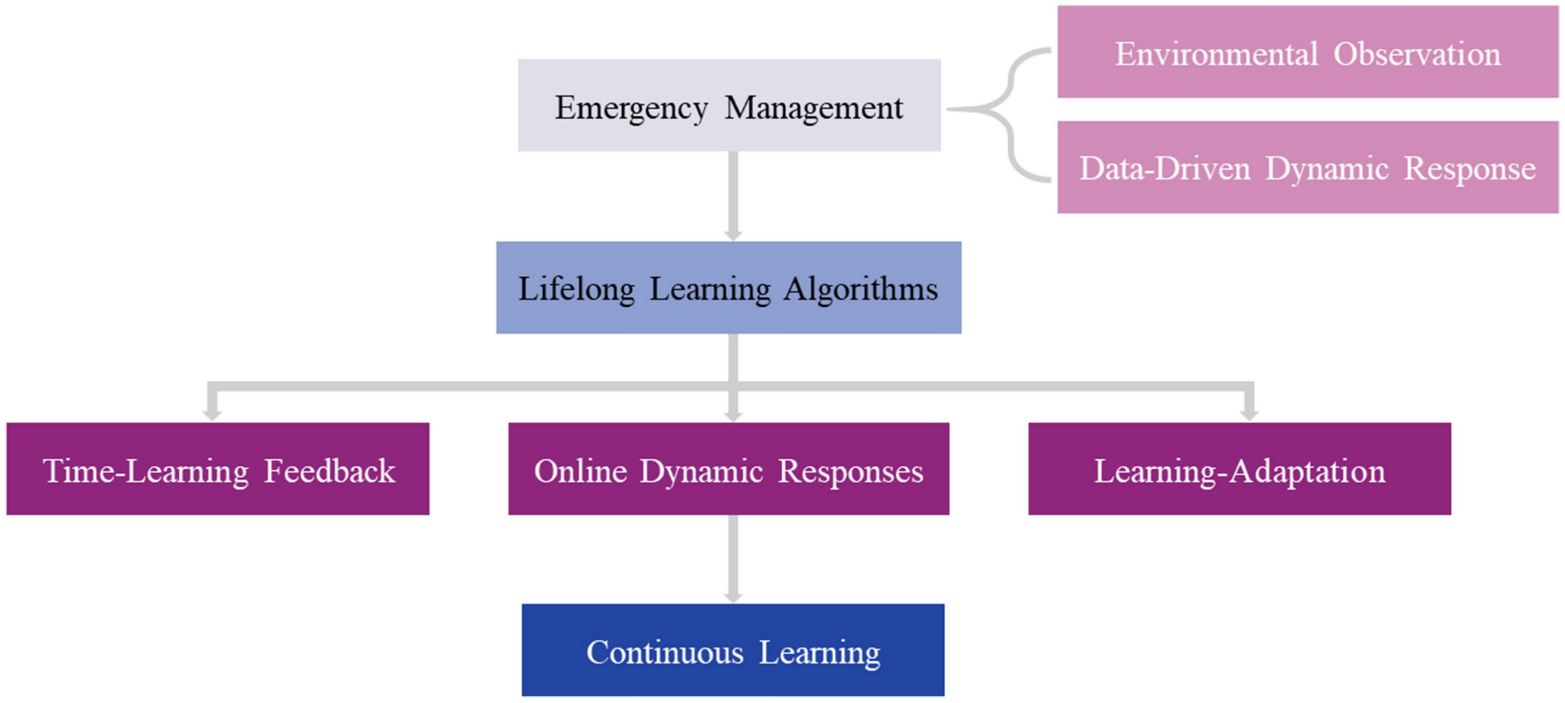
The emergency management process based on lifelong learning.

The purpose of emergency management is to provide autonomous agents with the ability to respond to unforeseen situations that are different from what they are trained or designed to handle. Therefore, a lifelong data-driven response-generation system must be developed to tackle this problem. It enables an agent to handle new scenarios without depending on the reliability of pre-existing models, safe states, and recovery strategies created offline or from prior experiences, or on their accuracy. The main finding is that, when needed, uncertainty in environmental observations may be used to inform the creation of quick, online reactions that effectively avoid threats and allow the agent to carry on operating and learning in its surroundings (Maguire et al., 2022). As is shown in Figure 6, the core process of emergency management has a close relationship with lifelong learning algorithms, it keeps learning and adapting.

## 4 Outlook

Lifelong learning with AIS has made significant progress in recent decades. And graph lifelong learning is emerging as an important area in AI research and applications. Graph lifelong learning involves applying lifelong learning principles to graph-based data structures and algorithms. This approach aims to enable systems to continuously learn and adapt from a stream of graph data over time. There are many kinds of graph lifelong learning algorithms, and there exist several differences between these methods, which suit different situations. Each method may have its own approach and principle to cope with problems, as can be seen in Table 1.

**Table 1:** Graph lifelong learning method comparison.

Methods	Approach			
	Architectural	Rehearsal	Regularization	Reference
Feature Graph Networks	Yes	No	No	Sarlin et al. (2020) and Zhou et al. (2022)
Hierarchical Prototype Networks	Yes	No	No	Li et al. (2023) and Zhang et al. (2023a)
Experience Replay GNN Frame work	No	Yes	No	Ahrabian et al. (2021a) and Zhou and Cao (2021b)
Lifelong Open-world Node Classification	No	Yes	No	Galke et al. (2021) and Zhang et al. (2022)
Disentangle-based Continual Graph Representation Learning	No	No	Yes	Kou et al. (2020) and Zhang et al. (2023b)
Graph Pseudo Incremental Learning	No	No	Yes	Tan et al. (2022) and Su et al. (2023)
Topology-aware Weight Preserving	No	No	Yes	Natali et al. (2020) and Liu et al. (2021)
Translation-based Knowledge Graph Embedding	No	No	Yes	Yoon et al. (2016) and Li et al. (2023)
Continual GNN	No	Yes	Yes	Han et al. (2020) and Wang et al. (2020)
Lifelong Dynamic Attributed Network Embedding	Yes	Yes	Yes	Li et al., 2017, Yoon et al. (2017), and Liu et al. (2021)

The key challenge in graph lifelong learning is to efficiently update and refine the model as new data arrives, without forgetting previously learned information (Galke et al., 2023). In addition, dynamic nature of graphs also brings problems for the reasons that graph data is often dynamic, such as social networks or knowledge graphs. Models need to adapt to these changes while maintaining the validity of past learning (Galke et al., 2023). Graph lifelong learning is a rapidly growing field that proposes new solutions for how intelligent systems can continuously learn and adapt to changing environments. With further research, this field is expected to solve existing challenges and provide strong support for the continued development and application of intelligent systems.

Besides the development of graph lifelong learning, several trends and directions can be observed in the relationship between lifelong learning algorithms and AIS. Firstly, multi-modal learning will play a crucial role as autonomous systems learn from diverse sensors and data sources, including visual, auditory, textual, and sensor data. This integration will greatly enhance the system’s perception and understanding capabilities. Secondly, an important aspect is self-improvement learning, where the system autonomously assesses its performance, identifies weaknesses, and automatically adjusts and improves its algorithms and models to enhance efficiency and accuracy. Furthermore, cross-domain transfer of knowledge and experience becomes a possibility. The system will be able to transfer learned knowledge from one domain to another, thereby enhancing its problem-solving abilities across different domains. What is more, lifelong learning with AIS can also be developed and applied in the area of education, especially in English teaching and learning. According to Grand View Research, the AI market in education is expected to reach $13.3 billion by 2025. Its diversity is able to change the form of language education to a certain extent, making it continuously transform from the original, traditional, and monotonous form to a dimensional, dynamic, and multi-spatial form, providing a personalized learning experience based on individual needs and preferences (Hwang et al., 2020). Although there has been little research on how lifelong learning can enhance English teaching and learning through AIS so far, it can benefit this area without doubt (Gao, 2021; Pikhart, 2021; Klimova et al., 2022).

Concerning lifelong learning algorithms themselves, incremental learning should receive more attention. Improving the efficiency and stability of incremental learning becomes crucial, enabling the system to retain previous knowledge while learning new tasks. Additionally, self-supervised learning methods will gain prominence. These techniques allow systems to learn from unlabeled data, reducing reliance on extensive labeled data and opening up opportunities for continuous learning. Overall, these trends and directions highlight the importance of multi-modal learning, self-improvement learning, cross-domain transfer, efficient incremental learning, and self-supervised learning in advancing the field of lifelong learning algorithms for AIS.

## 5 Conclusion

In this paper, we have extensively discussed the relationship between lifelong learning algorithms and autonomous intelligent systems. We have demonstrated the specific applications of lifelong learning algorithms in various domains such as autonomous driving, anomaly detection, service robotics, and emergency management. It is found that current research has made certain progress in addressing the catastrophic forgetting problem of complex scenarios and multitasking under long time sequences. However, challenges such as activation drift, inter-task confusion, and excessive neural resources still persist. In light of this, we particularly emphasize the significance and potential of advancing lifelong learning through graphical approaches, while pointing out that multimodal learning and methods like cross-domain transfer are pivotal references for future advancements in AIS lifelong learning algorithms. Among these, the integration of robot vision and tactile perception is recognized as a key challenge to enhance robot performance and efficiency. To conclude, lifelong learning proves to be a reliable and efficient method for advancing autonomous intelligent systems. Future research efforts should focus on developing fully autonomous and secure learning frameworks that offer superior performance while reducing the need for excessive supervision, training time, and resources.

## Author contributions

DZ: Conceptualization, Methodology, Resources, Writing – review & editing. QB: Investigation, Visualization, Writing – original draft. ZZ: Conceptualization, Funding acquisition, Methodology, Resources, Writing – review & editing. YZ: Investigation, Visualization, Writing – original draft. ZW: Funding acquisition, Resources, Writing – review & editing.
